# An innovative model of psychological service delivery in primary healthcare: the Single-Session Intervention

**DOI:** 10.1186/s12875-022-01949-8

**Published:** 2023-01-02

**Authors:** Kathy Perreault, Mylaine Breton, Djamal Berbiche

**Affiliations:** 1 Groupe de Médecine Familiale Universitaire de Saint-Jean-Sur-Richelieu, Saint-Jean-Sur-Richelieu, QC Canada; 2 Department of Family Medicine and Emergency Medicine, Faculty of Medicine and Health Sciences, Université de Sherbrooke, Sherbrooke, QC Canada; 3 Department of Community Health Sciences, Faculty of Medicine and Health Sciences, Université de Sherbrooke, Sherbrooke, QC Canada

**Keywords:** Single-session, Mental health, Primary care, Advanced access, Psychological consultation

## Abstract

**Background:**

In Canada, the demand for mental health care exceeds the provision of services. This exploratory study aimed to assess the feasibility and impact of a new service delivery model for psychological consultations in primary care settings: the Single-Session Intervention (SSI), inspired by Advanced Access (AA) principles for appointment scheduling. The specific objectives were to examine whether the SSI increases accessibility to psychological consultations, to measure the effects of the intervention on different self-reported measures, and to assess users’ consultation experiences.

**Methods:**

Participants were recruited in a University Family Medicine Group in Quebec (Canada), and the SSI was delivered by the on-site psychologist. No referral or formal diagnosis was needed to attend, and participants could promptly obtain an appointment. Participants rated the intensity of their problem, their level of psychological distress and their well-being, before and after the SSI. They also rated their satisfaction with their consultation experience. There was a follow-up 4 to 6 weeks later.

**Results:**

Of the *N* = 69 participants who received SSI, 91% were able to obtain an appointment in less than 7 working days. The number of patients who were able to benefit from a psychological consultation was about 7 times higher after the implementation of SSI compared to previous years, when a traditional model of service delivery was in place. After SSI, participants felt that the intensity of their problem and psychological distress were lower, and that their well-being was increased, as indicated by significant pre-post test clinical measures (*p* < 0.0001). The observed effects seemed to be sustained at follow-up. Moreover, 51% of participants said that one session was sufficient to help them with their problem. Participants rated SSI as a highly satisfying and helpful consultation experience (92,9% overall satisfaction).

**Conclusions:**

SSI, offered in a timely manner, could be an innovative and cost-effective intervention to provide mental health services on a large scale in primary healthcare. Further research is needed to replicate the results, but these preliminary data seem to indicate that psychological distress may be quickly addressed by SSI, thereby preventing further deteriorations in patients’ mental health.

**Trial Registration:**

2019–393, 26 March 2019.

## Background

In primary care settings in developed countries, depression and anxiety rank as the third most common reason for consultation [1]. Depression and anxiety are also considered the second most common condition in patients with comorbidities [2]. In Canada and throughout the world, mood and anxiety disorders are the most prevalent mental illnesses [3]. Individuals experiencing psychological distress most often initially consult in primary healthcare because it is accessible, less stigmatising, and more comprehensive, managing both physical and mental health [4]. In the previous decade, patients consulting for mental disorders accounted for 20 to 25% of family physicians’ clientele [5], but this number is likely much higher now. Indeed, a pan-Canadian survey, conducted during the pandemic, showed that 76% of primary care clinicians reported that they were seeing an increased number of patients with mental or emotional health needs [6].

In their mental health practices, primary care physicians mentioned that the major obstacles to care for patients are difficulty accessing specialised resources and the scarcity of mental healthcare workers [5]. They identified direct communication and collaboration with psychologists as being beneficial for patients [5]. Thus, having a psychologist integrated into a primary care team facilitates patient access to psychological services [7]. It is estimated that the integration of psychotherapy into primary care could result in a 20 to 30% decrease in medical costs [8]. However, limited availability of publicly funded services represents a real barrier to accessibility [9], leading to the creation of waiting lists that may stretch over several months [10].

The current COVID-19 pandemic has the potential to create a secondary crisis of psychological distress and mental health system spillover [11] that will have an increasingly negative impact on the mental health of communities [12]. Considering that current service delivery models are insufficient to meet this increased demand, innovative models are needed to deliver mental health care to communities [13]. The aim of this study is to analyze one such innovative model: a single-session intervention in psychology, delivered in primary healthcare.

### Choosing a model of service delivery: the Single-Session Intervention (SSI)

The primary care environment under study aims to provide mental health services, while fulfilling its mission to serve all enrolled patients and maintain quality of care. Previous experience has shown that traditional models of service delivery in psychology, offering 8 to 12 sessions for each patient (in short-term approaches), quickly leads to a blocking of the schedule and the creation of a waiting list of several weeks. With very limited resources (half-time psychologist), a new model of service delivery was required to respond to the needs of patients with psychological distress in a timely manner.

A review of the literature revealed that, when considering interventions in mental health, patients were generally very satisfied with brief treatment episodes, even with a single session [14]. In fact, the Single-Session Intervention (SSI) is not a new type of intervention, having been used by clinicians since the late 1970s [15, 16], but its recognition by the literature is more recent [17].

SSI is a modality of treatment that is conceptualized as an attitude or an approach to practice, rather than a specific therapeutic model [18, 19]. Considering that most users only come once [20] and that the majority of those attending an SSI find it sufficient and helpful [21], delivery of psychological services through SSI could make it possible to meet the mental health needs of many patients.

Even if SSIs may be seen as an “extreme” form of brief therapy [14], their short-term effectiveness has been shown by several studies [14, 21–24]. In addition, positive results and satisfaction levels seem to be maintained over the medium to long term, ranging from a few weeks to several years, both in adults and in children and adolescents [14, 23, 25, 26]. In terms of improving distress or initial symptoms, or reducing the severity of the problem/symptoms, effectiveness rates vary from 63 to 78% [17, 22, 23, 27, 28]. Similarly, other studies have shown a significant decrease in anxiety, depression, levels of distress, and psychopathology [29–32]. Moreover, the beneficial effects of an SSI are only slightly lower than those of a traditional multi-session intervention [33].

Considering that readiness for change is optimal when a person asks for help [16], as they are actively seeking to solve their problem [28], it is important that an intervention is delivered in a timely manner. Knowing that a delay in accessing services increases the risk of a person’s condition deteriorating [34] and their use of critical care services [35], Advanced Access (AA) model principles could inspire the provision of psychological services in primary healthcare [36]. AA is an organizational model that aims to improve accessibility for patients and support their relational and informational continuity with a primary healthcare provider or team [37]. The AA model is a patient-centered approach, where the patient can access the service they need from the right professional at the right time [38].

Initially developed in the United States in 2001, AA was mainly geared towards physicians and nurse, but it now seems relevant to engage in more interprofessional collaboration [39]. The AA models have been revisited to more interdisciplinary team practice, based on five guiding pillars; 1) comprehensive planning for needs, supply, and recurring variation, 2) regular adjustment of supply to demand, 3) processes of appointment booking and scheduling, 4) integration and optimization of collaborative practice, 5) communication about advanced access and its functionalities [40].

Since its development, AA has been implemented widely in North America, Europe, and Australia to engage professionals in more interprofessional collaboration [41, 42] and to ensure both effective implementation and patient-centered care [43]. A recent study exploring the possibilities and adaptations needed to transpose the principles of AA to a wider range of professionals [44] served as inspiration for the conceptualisation of an SSI in psychology in primary healthcare settings.

Therefore, the main objective of the present study is to assess the feasibility and the effects of an SSI in psychology, delivered in primary healthcare settings. Regarding accessibility, the specific objectives are: 1) to assess whether the SSI service delivery model leads to a significant increase in accessibility, in terms of the number of patients who obtain a psychological consultation; 2) to evaluate whether the SSI model allows for faster access to service; and 3) to verify if the SSI model leads to a decrease in missed appointments. Regarding the clinical effects of the SSI, the objectives are as follows: 1) to assess whether the SSI contributes to the reduction of the intensity of problem and psychological distress, as perceived by patients, as well as the increase of their emotional well-being; 2) to examine if these effects are maintained over time; and 3) to evaluate patients’ satisfaction with their consultation experiences.

## Methods

Given the exploratory nature of the study, the design was a pre-post intervention using a convenience sample without a control group. In addition to data on accessibility, we also wanted to evaluate clinical outcomes, from before to after SSI.

The study was conducted from April to December 2019 in a University Family Medicine Group (UFMG) in the province of Quebec (Canada). UFMGs are interdisciplinary family medicine groups affiliated with universities that have a research and teaching mission for family medicine residents. Since 2015, the FMG model has integrated different categories of primary healthcare providers, including family physicians, nurse practitioners, nurses, social workers, and pharmacists, among others [45]. However, psychologists are a scarce resource, as only 63 psychologists are working in the 370 FMGs throughout Quebec [46].

The study took place in a UFMG serving approximately 10,000 patients, composed of 13 family physicians, 20 family medicine residents, nine nurses, two pharmacists, two social workers, and one psychologist. The SSI under study was delivered by the team psychologist. The intervention was offered to all patients of the UFMG by every healthcare provider deeming it relevant. Information was given, and an appointment could be made on the spot. A leaflet could also be given to patients, with the option to call for an appointment later if needed. As for availability of the SSI, 10 consultation time slots were available each week, and the schedule was opened one week at a time. Patients were informed of the possibility to participate in the study just before their consultation, and they were given the choice to participate, with psychological consultations being delivered either way.

The inclusion criteria included all adult patients registered in the UFMG. However, patients in crisis (suicidal or homicidal ideation, psychotic crisis), struggling with problems of domestic violence, or involved in youth protection procedures were excluded. The consent form for the study was given at arrival by the receptionist, and patients were invited to read it while in the waiting room. Once in the psychologist’s office, the patient was asked whether they wished to participate in the study. If so, additional explanations were given as needed, and the consent form was signed.

The study was approved by three different review boards (Ethics, Research, and Institutional Suitability) within the CISSS de la Montérégie-Centre (project no. 2019–393).

### Description of the intervention (SSI)

SSI is an intervention addressing psychosocial issues that takes place in a single encounter, and is considered a complete intervention process in itself, with a beginning, a middle and an end [22]. There is no scheduled follow-up meeting, nor any subsequent appointments. The key aspect is to ensure that the patient leaves the session with a problem-solving plan and the confidence in their skills and resources to work through it, knowing that they can return at any time [18]. The active ingredients of an SSI are believed to be the same as the “common elements” ensuring success in psychotherapy, namely a strong emotional bond between the patient and the therapist, a confidential setting, an expectation of change, and a psychological explanation for the patient’s emotional distress that is deemed meaningful [47].

In the context of this study, the SSI lasted about 60 min, excluding the time spent obtaining informed consent and completing the pre-test questionnaires. A summary of the broad content of the intervention is provided in Table 1.

**Table 1:** Description of the SSI

Parts	Duration	Description
1. Development of the therapeutic alliance and validation of suffering	15 min	Explanation of the SSI. Empathetic presence and attentive listening. Recognition and validation of the distress felt. Identification of vulnerability to suicide and risk assessment
2. Definition of the problem and development of possible solutions or new perspectives	30 min	Focused discussion on the most important subject for the person. Co-construction of a plan with practical solutions or a summary of significant elements of the discussion
3. Co-editing of a personalized document and conclusion of the appointment	15 min	Co-drafting the document. Reminder of the person's strengths, resources, and successes. Establishment of protective measures/safety nets, if necessary. Details on how to obtain an additional SSI if needed

## Measures

Data about accessibility were collected using the electronic medical record and the clinic’s schedule. We collected the number of appointments booked and attended, and the number of different patients who have accessed SSI. Moreover, to evaluate timely access, one question concerning the delay between the need for consultation and the SSI itself was asked on questionnaires completed by participants. Sociodemographic data and clinical scales were also collected using paper questionnaires, and follow-up data was collected by phone 4 to 6 weeks later. We also used three clinical measures to assess SSI possible outcomes. At each step, questionnaires were placed in a sealed envelope and stored in a secured space and were only opened at the end of the experimentation period.

Table 2 shows these measures in more details.

**Table 2:** Measurement times, variables used, and brief descriptions

Time of measure	Measured Variables	Description	Scoring
T0Before SSI	Sociodemographic data	Standard questionnaire	N/A # of days Yes or No
	Two specific questions	1) Delay in obtaining SSI 2) First consultation	
	PPI	10-pt Likert Scale	1 to 10
	K6	5-pt Likert Scale (0 to 4)	Score: 0–24
	WHO-5	6-pt Likert Scale (0 to 5)	Score: 0–25 (× 4 = %)
T1 After SSI	PPI; K6; WHO-5 + one specific question Consulting Experience Questionnaire	See above Sufficiency of SSI 5-pt Likert Scale (5 sentences)	See above Yes or No Score: 0–5 (×20 = %)
T2 Follow-up	PPI; K6; WHO-5	See above	See above

*K6* Kessler Psychological Distress Scale–6 items, *PPI* Perceived Problem Intensity, *SSI* Single-Session Intervention, *WHO-5* World Health Organization Well-Being Index

In addition to the Likert Scale measuring Perceived Problem Intensity (PPI),  two validated clinical measures were used: the Kessler Psychological Distress Scale–6 items (K6), and the World Health Organization Well-Being Index (WHO-5). The K6 measures the presence of several nonspecific psychological distress symptoms relating to behaviors (e.g. restlessness or inability to sit still), emotions (e.g. nervousness, hopelessness), and cognition (e.g. feeling worthless or that everything is an effort). The scale is frequently used as a screening tool in population-level health surveys and has strong psychometric properties and good discriminating power between cases and “non-cases” of mental disorder [48, 49]. Scores above 13 are indicative of a severe mental health disorder, whereas scores of 0 to 12 indicate no such disorder, with a classification accuracy of 0.92 [48].

The WHO-5 is a valid measure of well-being in patients in primary care [50] that has good criterion validity for screening in this population [51]. Its psychometric properties are adequate: sensitivity ranges from 89 to 94%, and specificity ranges from 63 to 71% [50, 52, 53]. It is a self-reported measure of five items, all phrased positively, which cover positive mood (good mood, relaxed), vitality (being active and waking up refreshed), and interest in general (interested in several things). The raw score is multiplied by 4 to obtain a final score between 0 and 100, allowing for quantification of changes in well-being [54], with a threshold of 10% representing a clinically meaningful change [55].

In addition to these measures, the Consulting Experience Questionnaire was created to capture patients’ experiences and satisfaction with the SSI. This was done by amalgamating and adapting two existing scales, the Session Rating Scale [56] and the Single-Session Impressions & Feedback Tool (SSIFT)—Adapted Version, used by the Sudbury Child and Family Center [57]. Six questions on feeling respected and understood, being a partner in the resolution of problems, being helped by the session, and having developed hope, were organized on an Osgood’s Semantic Differential Scale. Respondents rated their perceptions by choosing the number closest to the sentence of their choice, the most negative statement being rated 1 and the most positive being rated 5. Their answers were then multiplied by 20 to obtain a percentage of satisfaction.

In terms of statistical analysis, the effects of the SSI were measured using analysis of variance (ANOVA) with repeated measures. To account for correlations between observations from the same individual, as multiple time points were used as repeated measures, the linear mixed model (LMM) with SAS PROC MIXED was used, this model being a generalization of a paired data model or a repeated measures ANOVA. In multivariable analysis, covariates can be measured once per individual (gender, for example) or repeatedly at each time point. One of the strengths of the LMM model is that it includes all measures for an individual even if some are missing for a given time point, whereas typical procedures eliminate individuals with incomplete responses. Thus, the structure of the data increases the power of the study because the analyses consider the number of valid observations rather than the number of participants [58]. We used an alpha of 0.0001 and a Bonferroni correction and only rejected the null hypothesis of each comparison if it had a p-value less than alpha/2 = 0.025.

## Results

### Participants

During the 8 months of the study, 129 patients received psychological consultations. Of these, 12,4% were children and adolescents and therefore, not included in the study. As for 7,9% of adult patients presenting for SSI, the psychologist delivering the intervention judged that their needs exceeded what could be offered within a single intervention. They were not included in the study, but still received psychological services. Another 7,7% of adult patients were excluded from the study because of their mental state when they consulted (perceptible cognitive impairment, active suicidal crisis or domestic violence that had to be reported to the Youth Protection Department). A total of 33 patients were excluded from the study but all benefited from psychological services.

Therefore, 96 patients were eligible for the study and 75% agreed to participate. A total of 72 participants were enrolled. Data from 3 participants were removed from the study since they came back each and every week. They were offered traditional psychotherapy by the on-site psychologist.

Study data is thus based on 69 enrolled participants. Table 3 presents their characteristics.

**Table 3:** Participant Demographics (*N* = 69)

Variable	%
Gender Identity	
Female	73
Male	27
Age (years)	
18–34	33
35–54	35
55–64	21
> 65	11
Education	
No diploma	14
High school diploma	16
Trade school studies	22
College graduate	21
University graduate	27
Family income (in CDN$)	
< 24 999	24
25 to 49 999	28
50 to 69 999	14
70 to 99 999	16
> 100 000	18
Marital status	
Single	23
Married/common-law	64
Separated/divorced	12
Widowed	1

As shown in Table 3, patients with very different characteristics accessed the SSI. For example, whereas a consultation rate of 12–17% is generally expected among men [59], it was 27% in the present study. Diversity was also observed in age groups, education, and family income.

### Accessibility

One objective of this study was to assess whether implementation of the model increased accessibility of psychological services. To evaluate this, we look at how many different patients had access to psychological consultations in the UFMG.

In the years 2016 and 2017, only 17 and 16 patients (respectively) benefited from psychological consultations. The year 2018 was particular, given the preparation for the SSI project. From January to September, a traditional model of service delivery in psychology was in place and 10 patients received psychological consultations. From the beginning of October, and until the end of December, there was a running-in period to assess the feasibility of the SSI. This service delivery model allowed 44 patients to access psychological consultations during this period.

As for 2019, the data show that during the 8-month period of the study, a total of 129 patients accessed psychological consultations through the SSI. These data confirmed that accessibility to psychological consultation, in terms of the number of patients benefiting from the service has indeed increased. Implementation of SSI allowed about 7 times more patients to access psychology consultations, when compared to previous years using traditional service model.

### Access in a timely manner

Another specific objective of the study was to verify whether AA principles of timely access would work with the SSI.

The new model of services allowed 91% of participants to obtain an appointment in fewer than 7 working days, and only 1% waited for more than 10 days. It is noteworthy that 22% received an appointment in less than one day, or on the same day as their consultation request.

### Impact on missed appointments/late cancellations

We also wanted to assess if the SSI would impact the no-show/late cancellation rate, which monopolize available clinical time and thus reduce access for potential new patients.

The no-show/late cancellation rate in previous years (2016 to 2018), with traditional service model, was about 22%. Implementation of SSI in 2019 seemed to have reduced this rate since it was at 10,4% during the study period.

### Sufficiency of a single session

We wanted to know if participants felt that a single session was enough for them to help solve their problem.

When asked, 51% of participants said that it was. Of those who visited more than once, 50% came twice, and another 50% came thrice, usually for the same problem stated during the first SSI. In the 8-month study period, only 19% of participants came more than once.

### Clinical outcomes of SSI

Although the study was observational in nature and the design did not include a control group, we were still interested in gathering data on clinical outcomes. These data should be interpreted with caution, as the study design is not powerful enough to ensure observed changes were attributable to the intervention.

Regarding the objectives related to the clinical effects of the SSI on the participants, the data suggested positive effects of the intervention (see Table 4).

**Table 4:** Means and standard deviations for measurement indicators of the SSI

Variable	Mean (SD)	F	df	*Cohen’s d*	*p*
***PPI***					
T0 – T1 (*n* = 82)	7.8 (1.7) vs 6.7 (2.2)	32.32	67	0.89	< 0.0001
T1 – T2 (*n* = 82)	6.7 (2.2) vs 6.9 (2.5)	0.18	59	0.07	0.610
T0 – T2 (*n* = 64)	7.8 (1.7) vs 6.9 (2.5)	11.89	60	0.58	0.0003
***K6***					
T0 – T1 (*n* = 82)	13.6 (4.5) vs 8.8 (4.5)	94.17	67	1.51	< 0.0001
T1 – T2 (*n* = 82)	8.8 (4.5) vs 8.7 (4.5)	0.10	59	0.05	0.798
T0 – T2 (*n* = 64)	13.6 (4.5) vs 8.7 (4.5)	72.15	60	1.42	< 0.0001
***WHO-5***					
T0 – T1 (*n* = 82)	39.3 (20.1) vs 57.9 (22.4)	73.91	67	1.34	< 0.0001
T1 – T2 (*n* = 82)	57.9 (22.4) vs 49.4 (21.3)	8.76	59	0.49	0.001
T0 – T2 (*n* = 64)	39.3 (20.1) vs 49.4 (21.3)	23.23	60	0.80	< 0.0001

*K6* Kessler Psychological Distress Scale–6 items, *PPI* Perceived Problem Intensity, *SSI* Single-Session Intervention, *WHO-5* World Health Organization Well-Being Index

For the first clinical outcome, we measured the participants perceived intensity (PPI) of their problem, by self-rating from 1 to 10, 10 being the “worst problem of their live”. When comparing T0 (before SSI) with T1 (immediately after SSI), results showed a significant reduction in PPI (7.8 vs 6.7, F = 32.32, df = 67, Cohen’s d = 0.89, *p* < 0.0001). To assess if the effect was sustained over time, we compare scores at T0 (before SSI) and at T2 (follow-up). We can see there is still a significant reduction in PPI at follow-up (7.8 vs 6.9, F = 11.89, df = 60, Cohen’s d = 0.58, *p* = 0.0003).

For the second clinical outcome, we measured participants’ psychological distress, with the K6. Compared to pre-SSI (T0), the distress scores also significantly decreased (13.6 vs 8.8, F = 94.17, df = 67, Cohen’s d = 1.51, *p* < 0.0001) after the intervention (T1). This reduction seemed to have been maintained at follow-up, when compared to pre-SSI (T0—T2) (13.6 vs 8.7, F = 72.15, df = 60, Cohen’s d = 1.42, *p* < 0.0001). Furthermore, before the intervention (T0), 49% of participants reached the K6 cut-off score (≥ 13 pts) indicating the probability of a serious mental illness [48]. After the SSI (T1), only 18% remained in this category.

As for the third clinical outcome, the well-being of participants was measured by the WHO-5 index. The results indicate a significant increase in well-being scores from before to after the SSI (T0—T1) (39.3 vs 57.9, F = 73.91, df = 67, Cohen’s d = 1.34, *p* < 0.0001). At follow-up, the increased well-being also seemed to be maintained, when compared to pre-SSI (T0 – T2) (39.9 vs 49.4, F = 23.23, df = 60, Cohen’s d = 0.80, *p* < 0.0001).

Interestingly, presumed effect sizes of the SSI, as measured by Cohen’s d [60], varies from moderate to large (0.58 to 1.81). Table 4 presents results in more details.

### Satisfaction with the SSI

Assessing the satisfaction of participants with the intervention was also an objective of the study. This was measured immediately after the intervention using the Consulting Experience Questionnaire, in which participants had to endorse a statement describing their opinion, with 1 being the least positive statement and 5 being the most positive.

As shown in Table 5, participants seemed to be mostly satisfied with their SSI experience, feeling they were understood, helped, and successful in solving their problems. Overall satisfaction with the consultation experience was 92.9%.

**Table 5:** Consulting experience questionnaire

**Endorsed statement**	**Mean and SD***N* = 82	**%**
I felt respected, listened to, and understood	4.98 (0.16)	99.6
We have covered the subjects I wanted to talk about	4.92 (0.28)	98.4
I felt that I was an important partner in the consultation	4.88 (0.53)	97.6
I was very active in solving my problem	4.40 (0.77)	88.0
I feel full of hope after this meeting	4.17 (0.83)	83.4
This meeting helped me a lot	4.52 (0.78)	90.4
**Overall mean of satisfaction**	4.65	92.9

## Discussion

Accessibility of psychological services in primary healthcare was the major concern that led to pilot this new service delivery model. As expected, patients in need of mental health services gained increased access with the implementation of the SSI, which was reinforced by the application of AA pillars related to short-term planning of the schedule to maintain availability for more urgent needs [44]. Indeed, most patients received their consultation in fewer than 7 working days, and almost a quarter received an appointment in less than a day. Moreover, the fact that the appointment could be obtained so close to the perceived need of the patient seemed to have reduced the no-show/late cancellation rate. This decrease in no-shows was also reported by the first physicians to implement advanced access in their practice [38].

As for the results of the intervention itself, the results seemed to indicate that, even if participants received only one psychological intervention, they felt that their presenting problem decreased in intensity, their psychological distress was reduced, and their well-being was augmented, these improvements being statistically significant. Furthermore, effects appeared to last over time, from 4 to 6 weeks after SSI. Finally, participants were very satisfied with their SSI consultation experience.

These results are in line with qualitative literature on SSIs, which indicated that clients found an SSI was reassuring and provided information, support, and hope, and that it helped them to clarify their difficulties, give new perspectives, and foster a more positive outlook [61]. The fact that half of participants perceived a single session as sufficient is consistent with other studies as well [22, 25, 26, 28, 29, 61, 62].

### Limitations

Despite these encouraging results, there were many limitations to this study. The first and foremost was that the clinician carrying out the SSI and the primary researcher were the same person. Even though mitigation measures were taken to ensure that bias was reduced to a minimum, studies with different professionals and in other settings need to be undertaken to replicate the results. A RCT with a pragmatic control, or a comparison with usual care, such as mental health consultation with a general practitioner, could be used. Moreover, the follow-up needs to be longer and consider what might have happened in patients live during this time (meaningful events, consultation of another professional, etc.).

Clearly, the use of a convenience sample study, with no comparison group, greatly limits the generalizability of the findings. Indeed, since there was no control group, the observed changes may not necessarily be attributable to the intervention. However, the aim of the study was exploratory, allowing for observations of the potential effects of a new model of delivery for psychological services (an SSI following AA scheduling principles) and assessing the feasibility of implementation in primary healthcare settings, without additional resources. Even with limitations related to the study design, the present study makes a unique contribution, demonstrating that it is possible to respond quickly to the mental health needs of patients with very little resources and investment. Importantly, this can be done in primary care settings, where people feel comfortable receiving healthcare, and a single session seems sufficient for half of patients. Additionally, the SSI's open-door approach, allowing patients to return as needed, provides a form of safety net. The service model also enables those with more important mental health needs to be identified and taken care of.

Currently, SSI services are offered in different countries (Australia, United States, United Kingdom) and in some Canadian provinces (Alberta, British Columbia, Newfoundland and Labrador, Ontario). However, to our knowledge, in the province of Quebec, there is no publicly funded government institution that provides an SSI from a population-based perspective in FMGs or other primary healthcare settings. This study demonstrates that an SSI could be implemented in Quebec’s primary healthcare services and have a real positive impact on patients, by responding in a timely manner to their mental health needs. Furthermore, there is substantial evidence in literature that non-professionals can deliver effective, sensitive, and acceptable care for diverse mental health challenges [63]. We believe that the SSI model of service, coupled with AA scheduling principles, would be an ideal model to enable expanded access to services for people with mental health issues.

## Conclusions

To maintain universal service coverage of frontline mental health care in a context of limited resources, service delivery models need to be different, even if it means being “disturbing” [20]. The SSI represents a “disturbing” innovation since it challenges the traditional mental health service delivery model. In doing so, SSI might be considered as an alternative that helps to respond to mental health needs of the population, in a simpler and less costly way [20].

A potential public criticism of SSI is that it represents a “service cut” due to its obvious briefness. However, rather than seeking to cut services, the SSI must be considered as an additional service, intended to provide immediate help to patients in psychological distress [64], thereby limiting the number of referrals to specialized mental health programs, which are already overloaded. SSI is seen as a part of a continuum of mental health services and it constitutes only one of its components [31], knowing that it is not suitable for all individuals or for all problems [65].

As mental health problems continue to rise in the never-ending COVID-19 pandemic, innovative models to deliver mental health support to communities are needed. By delivering a prompt and effective intervention, SSIs have the potential to meet the needs of many patients and to increase access to mental health services in a cost-effective manner [66]. When combined with AA scheduling principles, SSIs could constitute a useful addition to the continuum of mental health services that is accessible, easily replicable, and more sustainable, especially in the context of high demand and limited resources.

## Data Availability

The datasets generated and analyzed during the current study are not publicly available due to a risk of breach of confidentiality. They can be made available by the corresponding author on reasonable request.

## Abbreviations

AA: Advanced Access
ANOVA: Analysis of variance
FMG: Family Medicine Group
K6: Kessler Psychological Distress Scale–6 items
LLM: Linear mixed model
RCT: Randomized controlled trial
SSI: Single-Session Intervention
SSIFT: Single-Session Impressions & Feedback Tool
UMFG: University Family Medicine Group
WHO-5: World Health Organization Well-Being Index

## References

[1] Finley C, Chan D, Garrison S, Korownyk C, Kolber M, Campbell S (2018). What are the most common conditions in primary care? systematic review. Can Fam Physician.

[2] Cassell A, Edwards D, Harshfield A, Rhodes K, Brimicombe J, Payne R (2018). The epidemiology of multimorbidity in primary care: a retrospective cohort study. Br J Gen Pract.

[3] McRae L, O’Donnell S, Loukine L, Rancourt N, Pelletier C (2016). Report summary - mood and anxiety disorders in Canada, 2016. Health Promot Chronic Dis Prev Can Res Policy Pract.

[4] Rothman AA, Wagner EH (2003). Chronic illness management: what is the role of primary care?. Ann Intern Med.

[5] Fleury M-J, Imboua A, Aubé D, Farand L, Lambert Y (2012). General practitioners’ management of mental disorders: a rewarding practice with considerable obstacles. BMC Fam Pract.

[6] Wong S, Network TP and IHCI, Research TCI for HRS for PO, Nova Scotia Health Authority D of B and DNS, Advisory Group LAGC. Quick COVID-19 Primary Care Survey of Clinicians: Summary of the sixth (May 29-June 1, 2020) pan-Canadian survey of frontline primary care clinicians’ experience with COVID-19. (English and French) 2020.

[7] Miller-Matero LR, Dubaybo F, Ziadni MS, Feit R, Kvamme R, Eshelman A (2015). Embedding a psychologist into primary care increases access to behavioral health services. J Prim Care Community Health.

[8] Satterfield JM. Core Competencies of the Primary Care Provider in an Integrated Team. Behav. Health Prim. Care Effic. Eff. Rep. Third Reno Conf. Integr. Behav. Health Prim. Care, Reno, NV, US: Context Press/New Harbinger Publications; 2003, p. 45–68.

[9] Alhawshani S, Furmli S, Shuvra MMR, Malick A, Dunn LB, Ogrodniczuk JS (2019). Psychotherapy for patients with mental health concerns in primary care. Can Fam Physician.

[10] Fansi A, Jehanno C, Institut national d’excellence en santé et en services sociaux (Québec). Avis sur l’accès équitable aux services de psychothérapie. Québec, Qc: INESSS; 2015.

[11] Choi KR, Heilemann MV, Fauer A, Mead M (2020). A second pandemic: mental health spillover from the Novel Coronavirus (COVID-19). J Am Psychiatr Nurses Assoc.

[12] Lima CKT, Carvalho PM de M, Lima I de AAS, Nunes JVA de O, Saraiva JS, de Souza RI, et al. The emotional impact of Coronavirus 2019-nCoV (new Coronavirus disease). Psychiatry Res 2020;287:112915. 10.1016/j.psychres.2020.112915. 10.1016/j.psychres.2020.112915 PMC7195292 32199182

[13] Moreno C, Wykes T, Galderisi S, Nordentoft M, Crossley N, Jones N (2020). How mental health care should change as a consequence of the COVID-19 pandemic. Lancet Psychiatry.

[14] Kaffman M (1995). Brief therapy in the Israeli kibbutz. Contemp Fam Ther.

[15] Johnson NJ, Whitaker LC, Porter G (1980). The development and efficacy of a university mental health service walk-in clinic. J Am Coll Health Assoc.

[16] Schoener G (1977). No-red-tape counseling for clients alienated from traditional services. Psychiatr Serv.

[17] Talmon M (1990). Single-session therapy: Maximizing the effect of the first (and often only) therapeutic encounter.

[18] Campbell A (2012). Single-session approaches to therapy: time to review. Aust N Z J Fam Ther.

[19] Rycroft P, Young J (1997). Single session therapy: capturing the moment. Psychother Aust.

[20] Rotheram-Borus MJ, Swendeman D, Chorpita BF (2012). Disruptive Innovations for designing and diffusing evidence-based interventions. Am Psychol.

[21] Hymmen P, Stalker CA, Cait C-A (2013). The case for single-session therapy: does the empirical evidence support the increased prevalence of this service delivery model?. J Ment Health.

[22] Price C (1994). Open days making family therapy accessible in working class suburbs. Aust N Z J Fam Ther.

[23] Perkins R (2006). The effectiveness of one session of therapy using a single-session therapy approach for children and adolescents with mental health problems. Psychol Psychother Theory Res Pract.

[24] Schleider JL, Weisz JR (2017). Little treatments, promising effects? Meta-analysis of single-session interventions for youth psychiatric problems. J Am Acad Child Adolesc Psychiatry.

[25] Slive A, MacLaurin B, Oakander M, Amundson J (1995). Walk-in single sessions: a new paradigm in clinical service delivery. J Syst Ther.

[26] Perkins  R, Scarlett  G (2008). The effectiveness of single session therapy in child and adolescent mental health. Part 2: an 18-month follow-up study. Psychol Psychother Theory Res Pract.

[27] Hampson R, O’Hanlon J, Franklin A, Pentony M, Fridgant L, Heins T (1999). The place of single session family consultations: five years’ experience in Canberra. Aust N Z J Fam Ther.

[28] Miller JK, Slive A (2004). Breaking down the barriers to clinical service delivery: walk-in family therapy. J Marital Fam Ther.

[29] Harper-Jaques S, Foucault D (2014). Walk-in single-session therapy: client satisfaction and clinical outcomes. J Syst Ther.

[30] Campbell A (1999). Single session interventions: an example of clinical research in practice. Aust N Z J Fam Ther.

[31] Harper-Jaques S, McElheran N, Slive A, Leahey M (2008). A comparison of two approaches to the delivery of walk-in single session mental health therapy. J Syst Ther.

[32] Stalker CA, Horton S, Cait C-A (2012). Single-session therapy in a walk-in counseling clinic: a pilot study. J Syst Ther.

[33] Weisz JR, Kuppens S, Ng MY, Eckshtain D, Ugueto AM, Vaughn-Coaxum R (2017). What five decades of research tells us about the effects of youth psychological therapy: a multilevel meta-analysis and implications for science and practice. Am Psychol.

[34] Ordre des psychologues du Québec. Accès aux services psychologiques : un problème sous-estimé. https://www.ordrepsy.qc.ca/-/acces-aux-services-psychologiques-un-probleme-sous-estime/1.4.

[35] Collectif pour l’accès à la psychothérapie. La psychothérapie : un meilleur accès pour tous par des services assurés. 2015. https://capqc.ca/sites/capqc.ca/files/uploads/articles/2015/memoire-acces-services-psychotherapie.pdf.

[36] Ansell D, Crispo JAG, Simard B, Bjerre LM (2017). Interventions to reduce wait times for primary care appointments: a systematic review. BMC Health Serv Res.

[37] Murray M, Berwick DM (2003). Advanced access: reducing waiting and delays in primary care. JAMA.

[38] Breton M, Maillet L, Paré I, Abou Malham S, Touati N (2017). Perceptions of the first family physicians to adopt advanced access in the province of Quebec, Canada. Int J Health Plann Manage.

[39] Murray M, Tantau C (2000). Same-day appointments: exploding the access paradigm. Fam Pract Manag.

[40] Breton M, Gaboury I, Beaulieu C, Sasseville M, Hudon C, Malham SA (2022). Revising the advanced access model pillars: a multimethod study. CMAJ Open.

[41] Breton M, Maillet L, Duhoux A, Abou Malham S, Gaboury I, Manceau L (2020). Evaluation of the implementation and associated effects of advanced access in university family medicine groups: a study protocol. BMC Fam Pract.

[42] Murray M (2005). Answers to your questions about same-day scheduling. Fam Pract Manag.

[43] Tantau C (2009). Accessing patient-centered care using the advanced access model. J Ambulatory Care Manage.

[44] Gaboury I, Breton M, Perreault K, Bordeleau F, Descôteaux S, Maillet L (2021). Interprofessional advanced access – a quality improvement protocol for expanding access to primary care services. BMC Health Serv Res.

[45] Breton M, Lévesque J-F, Pineault R, Hogg W (2011). Primary care reform: can Quebec’s family medicine group model benefit from the experience of Ontario’s family health teams?. Healthc Policy.

[46] Ordre des psychologues du Québec. Mémoire de l’Ordre des psychologues du Québec dans le cadre des consultations concernant les effets de la pandémie sur la santé mentale. 2021. https://www.ordrepsy.qc.ca/c/document_library/get_file?uuid=5ad34f11-f5db-da16-b13f-e401b6ced726&groupId=26707

[47] Al-Khatib B, Norris S (2015). A family consultation service: single session intervention to build the mental health and wellbeing of children and their families. Educ Child Psychol.

[48] Kessler RC, Barker PR, Colpe LJ, Epstein JF, Gfroerer JC, Hiripi E (2003). Screening for serious mental illness in the general population. Arch Gen Psychiatry.

[49] Kessler RC, Andrews G, Colpe LJ, Hiripi E, Mroczek DK, Normand S-L (2002). Short screening scales to monitor population prevalences and trends in non-specific psychological distress. Psychol Med.

[50] Henkel V, Mergl R, Kohnen R, Maier W, Möller H-J, Hegerl U (2003). Identifying depression in primary care: a comparison of different methods in a prospective cohort study. BMJ.

[51] Löwe B, Spitzer RL, Gräfe K, Kroenke K, Quenter A, Zipfel S (2004). Comparative validity of three screening questionnaires for DSM-IV depressive disorders and physicians’ diagnoses. J Affect Disord.

[52] Mergl R, Seidscheck I, Allgaier A-K, Möller H-J, Hegerl U, Henkel V (2007). Depressive, anxiety, and somatoform disorders in primary care: prevalence and recognition. Depress Anxiety.

[53] Saipanish R, Lotrakul M, Sumrithe S (2009). Reliability and validity of the Thai version of the WHO-Five Well-Being Index in primary care patients. Psychiatry Clin Neurosci.

[54] Blom EH, Bech P, Högberg G, Larsson JO, Serlachius E (2012). Screening for depressed mood in an adolescent psychiatric context by brief self-assessment scales – testing psychometric validity of WHO-5 and BDI-6 indices by latent trait analyses. Health Qual Life Outcomes.

[55] Topp CW, Østergaard SD, Søndergaard S, Bech P (2015). The WHO-5 Well-being index: a systematic review of the literature. Psychother Psychosom.

[56] Duncan B, Miller S, Sparks J, Claud D, Reynolds L (2003). The session rating scale: preliminary psychometric properties of a “Working” alliance measure. J Brief Ther.

[57] Cooper S. Quality assurance at the Walk-in clinic: Process, outcome, and learning 2013:8.

[58] Yang J, Zaitlen NA, Goddard ME, Visscher PM, Price AL (2014). Advantages and pitfalls in the application of mixed model association methods. Nat Genet.

[59] SOM Sondage. Sondage auprès des hommes québécois habitant l’Ile de Montréal. Montréal, Québec: Comité régional en santé et bien-être des hommes de la région de Montréal; 2021.

[60] Cohen J. Statistical Power Analysis for the Behavioral Sciences. 2nd ed. New York: Routledge; 1988. 10.4324/9780203771587.

[61] Young K, Dick M, Herring K, Lee J (2008). From waiting lists to walk-in: Stories from a walk-in therapy clinic. J Syst Ther.

[62] Young K (2017). Narrative practice and the de-pathologizing of children’s lives at a walk-in therapy clinic: An opportunity for socially just conversations.

[63] Barnett ML, Lau AS, Miranda J (2018). Lay Health worker involvement in evidence-based treatment delivery: a conceptual model to address disparities in care. Annu Rev Clin Psychol.

[64] Bloom K, Tam JA (2015). Walk-in services for child and family mental health. J Syst Ther.

[65] Slive A, McElheran N, Lawson A (2008). How brief does it get? walk-in single session therapy. J Syst Ther.

[66] Bertuzzi V, Fratini G, Tarquinio C, Cannistrà F, Granese V, Giusti EM (2021). Single-Session therapy by appointment for the treatment of anxiety disorders in youth and adults: a systematic review of the literature. Front Psychol.

